# An Epidemiological Study of Concomitant Use of Chinese Medicine and Antipsychotics in Schizophrenic Patients: Implication for Herb-Drug Interaction

**DOI:** 10.1371/journal.pone.0017239

**Published:** 2011-02-16

**Authors:** Zhang-Jin Zhang, Qing-Rong Tan, Yao Tong, Xue-Yi Wang, Huai-Hai Wang, Lai-Ming Ho, Hei Kiu Wong, Yi-Bin Feng, Di Wang, Roger Ng, Grainne M. McAlonan, Chuan-Yue Wang, Vivian Taam Wong

**Affiliations:** 1 School of Chinese Medicine, Faculty of Medicine, the University of Hong Kong, Hong Kong, China; 2 Department of Psychiatry, Fourth Military Medical University, Xijing Hospital, Xi'an, Shaanxi, China; 3 Mental Health Center, Hebei Medical University, Shijiazhuang, Hebei, China; 4 School of Public Health, the University of Hong Kong, Hong Kong, China; 5 Department of Psychiatry, Kowloon Hospital, Hong Kong, China; 6 Department of Psychiatry, the University of Hong Kong, Hong Kong, China; 7 Capital Medical University, Beijing Anding Hospital, Beijing, China; 8 Chinese Medicine Section, Hospital Authority, Hong Kong, China; Pennsylvania State University, United States of America

## Abstract

**Background:**

Herb-drug interactions are an important issue in drug safety and clinical practice. The aim of this epidemiological study was to characterize associations of clinical outcomes with concomitant herbal and antipsychotic use in patients with schizophrenia.

**Methods and Findings:**

In this retrospective, cross-sectional study, 1795 patients with schizophrenia who were randomly selected from 17 psychiatric hospitals in China were interviewed face-to-face using a structured questionnaire. Association analyses were conducted to examine correlates between Chinese medicine (CM) use and demographic, clinical variables, antipsychotic medication mode, and clinical outcomes. The prevalence of concomitant CM and antipsychotic treatment was 36.4% [95% confidence interval (95% CI) 34.2%–38.6%]. Patients using concomitant CM had a significantly greater chance of improved outcomes than non-CM use (61.1% vs. 34.3%, OR = 3.44, 95% CI 2.80–4.24). However, a small but significant number of patients treated concomitantly with CM had a greater risk of developing worse outcomes (7.2% vs. 4.4%, OR = 2.06, 95% CI 2.06–4.83). Significant predictors for concomitant CM treatment-associated outcomes were residence in urban areas, paranoid psychosis, and exceeding 3 months of CM use. Herbal medicine regimens containing Radix Bupleuri, Fructus Gardenia, Fructus Schisandrae, Radix Rehmanniae, Akebia Caulis, and Semen Plantaginis in concomitant use with quetiapine, clozapine, and olanzepine were associated with nearly 60% of the risk of adverse outcomes.

**Conclusions:**

Concomitant herbal and antipsychotic treatment could produce either beneficial or adverse clinical effects in schizophrenic population. Potential herb-drug pharmacokinetic interactions need to be further evaluated.

## Introduction

With the widespread use of various herbal products which are often concomitantly used with pharmaceutical drugs, herb-drug interactions have become an important issue in drug safety and clinical practice [1], [2]. This was initially because of several case studies reporting nephrotoxic and hepatotoxic effects associated with herbal medicine use [3]–[5]. It is well documented that concomitant use of herbal medicine with conventional drug treatment can alter pharmacokinetic profiles of many classes of pharmaceutical drugs, including psychotropic agents, anticoagulants, oral contraceptives, immunosuppressants, cardiovascular drugs, anti-HIV, anticancer agents and antiepileptics [1], [2], [6]. However, whether such concomitant treatment with herbal and conventional medicine is associated with clinical outcomes in a defined population of patients remains to be determined.

Most patients with schizophrenia may develop a chronic course and are required for long-term maintenance treatment [7]. Although antipsychotic therapy is a mainstay in the maintenance treatment of schizophrenia, patients still often experience relapse and various adverse events caused by antipsychotic treatment [7]. In order to improve the therapeutic efficacy and reduce adverse side effects related to antipsychotic therapy, herbal medicine and other alternative therapies have been increasingly introduced into the treatment of schizophrenia [8]. This is particularly apparent in Chinese patients who have distinctive perceptions of Chinese medicine (CM), of which herbal materials account for about 85% preparations and products.

Numerous studies have shown therapeutic benefits of herbal medicine for persistent negative symptoms, cognitive impairment, and adverse side effects in schizophrenic patients [8]. Our recent study revealed that herbal medicine could alleviate hyperprolactinemia in schizophrenic patients [9]. Nevertheless, there are also case studies reporting acute and persistent psychosis caused by herbal supplementary use [10], [11]. These data suggest that herbal medicine in combination with antipsychotic drugs may produce either positive or negative clinical effects, making it important to examine potential relationships between clinical outcomes and concomitant treatment with herbal and antipsychotic agents in schizophrenia.

The primary objective of this epidemiological survey was to determine the prevalence of CM concomitant use and its associations with demographic, clinical characteristics and treatment outcomes in a random sample of patients with schizophrenia through face-to-face interview using a structured questionnaire.

## Methods

### Study design and setting

This retrospective, cross-sectional epidemiological study was conducted in 17 psychiatric hospitals and mental health centers in China. The selection of the study sites was based on geographic and sociodemographic variations as previously described [12], with particular consideration of regions, local economic development levels, and overall educational levels, as these variables are heavily related to perceptions and beliefs for CM. The study was approved by Institutional Review Board (IRB) of the University of Hong Kong/Hospital Authority Hong Kong West Cluster and registered at www.HKClinicalTrials.com (HKCTR-874). All participants or their guardians were required to give written informed consent for participating in the survey. The survey was conducted between April 2009 and September 2009.

### Population and sample

The study population was confined to patients with schizophrenia who visited psychiatric clinics or were hospitalized during the survey period. Patients who met the following criteria were eligible for the study: (1) aged 15 years or above; (2) had a primary diagnosis of schizophrenia or schizoaffective disorder based on *International Classification of Diseases (ICD)*, *10th Edition* [13]; (3) had been taking conventional antipsychotic treatment for at least 6 weeks; and (4) patients, their caregivers and/or doctors could provide necessary information about CM use if CM was used.

One key question that the present study attempted to answer was whether concomitant use of CM could affect the clinical outcomes of patients with schizophrenia, particularly adverse outcomes related to concomitant CM and antipsychotic use. Estimation of sample size was therefore based on the prevalence of CM use and the proportion of CM users with worse outcomes (see below). These two indices had been obtained from a pilot survey of 297 patients with schizophrenia [8], showing that nearly 36% of patients concomitantly used CM and 5.7% of CM users experienced worse outcomes, while only 2.8% of non-CM users had worse outcomes. In order to detect a 2.9% difference in the rate of worse outcomes between CM users and non-CM users, with a power (1-β) of 80% and a two-tailed level of α  = 0.05, 1750 participants were required to detect statistical difference in terms of worse outcomes associated with concomitant CM use. The number of surveyed subjects allocated to each study site was determined based on the volume of visits and annual admission numbers. The selection of eligible patients at each survey site was determined using random number tables.

### Study instruments

A specifically designed structured questionnaire was administered in the survey. The questionnaire covered: (1) sociodemographic and clinical characteristics; (2) purpose, advice source, attitude, and awareness of CM use; (3) the concomitant use pattern including individual CMs, conventional medication modes, and duration of the concomitant use; and (4) clinical outcomes. Chinese medicine (CM) is defined as preparations and products in powder, tablet, capsule, soft-gel, or liquid form prepared from single or mixed herbal, mineral, and animal materials or extracts [8]. CM users were defined as those who had been using CM consecutively for at least one month or cumulatively for at least 3 months with no more than 45 days of absence in total and no more than 7 consecutive days of absence when the survey was conducted. Those who never or only occasionally used CM were defined as non-CM users. This definition of the length of CM use was based on CM clinical practice, demonstrating that CM therapy of most chronic mental-emotional conditions requires a considerable period before observable improvements are achieved [14].

Clinical outcomes were classified as improved, worse, and unchanged condition. Improved outcomes were defined as clinically meaningful improvements occurring in the preceding one month on one or more conditions as follows: (1) psychosis; (2) comorbid psychiatric symptoms, mainly anxiety, depression, cognitive impairment, and sleep disorders; (3) adverse side effects associated with antipsychotic therapy, frequently body weight gain, constipation, enuresis, hyperprolactinemia, hypersalivation, leukopenia, and tardive dyskinesia; and (4) comorbid non-psychiatric conditions, such as hypertension and diabetes. Worse outcomes were defined as hospitalization, emergency room visits, or changes in medication modes due to the worsening of psychosis, comorbid symptoms, intolerable side effects, or the occurrence of new comorbid symptoms in the preceding one month. Patients who did not experience either clinically meaningful improvement or worsening in the preceding one month were defined as subjects with unchanged conditions. The assessment of clinical outcomes was conducted by psychiatrists based on changes in the severity and frequency of episodes of related symptoms, physical examination, and laboratory tests as well as reports from patients and their guardians.

### Survey procedures

The survey was performed by trained psychiatrists on site with face-to-face interview. To ensure consistency of the survey across sites and over time, two sessions of training workshop were conducted for interviewers who were practicing psychiatry. Upon completion of the training workshops, inter-rater reliability was assessed by calculating interrater agreement coefficients (κ value) for the designed questionnaire. All interviewers had achieved a κ value of at least 0.8 after training sessions. In addition, post-survey interviews were further conducted to verify missing and illogical data.

### Data analysis

The prevalence of CM use was calculated using maximum likelihood estimation of logistic regression. Chi-square (χ^2^) test was used to determine bivariate associations between CM use and demographic and clinical variables. Binary logistic regression model was further used for multivariate analyses to identify independent factors associated with CM use from the same variable tested in the bivariate analysis. The association between clinical outcomes and CM use was also examined using Chi-square (χ^2^) test and binary logistic regression analysis, with adjustment for demographic and clinical variables that were found to be significantly associated with CM use.

Subgroup analyses were conducted in CM-using subjects to further determine associations between clinical outcomes and CM and antipsychotic concomitant use modes. Chi-square test and multinomial logistic regression model were respectively utilized to examine bivariate and multivariate associations of clinical outcomes with demographic and clinical variables. Multinomial logistic regression model was also applied to evaluate associations of clinical outcomes with individual CMs and antipsychotic regimens that were used in at least 5% of CM-using respondents with either improved or worsened conditions, with adjustment for demographic and clinical variables that were shown to be significantly associated with clinical outcomes.

Odds ratio (OR) and 95% confidence interval (95% CI) were obtained from binary and multinomial logistic regression analysis. In association analyses of clinical outcomes, the unchanged outcome served as reference for improved and worse outcome in the calculation of OR values. All analyses were performed with SPSS version 16 software (Chicago, IL, USA). Statistical significance was defined as p<0.05 and all tests were two-sided.

## Results

### The characteristics of the sample

In a total of 1795 eligible subjects surveyed, seven were excluded from the analyses due to missing basic demographic and clinical data (gender, age, and the illness duration). In the remaining 1788 subjects who were included in the final analyses, 51% were males and the mean (±SD) age was 32±12 years. Fifty-three percent were diagnosed with paranoid schizophrenia. These demographic and clinical characteristics were similar to those reported in previous epidemiological surveys of schizophrenic population in China [15].

### The characteristics of antipsychotic medication modes

Thirty-five psychotropic drugs were identified. Medication regimens of 99.3% subjects included antipsychotics and 43% had two or more antipsychotics. The remaining 0.7% subjects were medicated with mood stabilizers occasionally combined with antipsychotics. The most commonly used antipsychotics in all subjects were risperidone (50.8%), quetiapine (21.0%), clozapine (17.2%), olanzapine (8.2%), and phenothiazines (7.0%). This medication mode was similar to that previously reported in China [16]. No significant differences were observed in frequency distribution of these antipsychotic regimens between CM- and non-CM-using subjects (see below), except for risperidone monotherapy. For the latter the proportion of CM-using respondents treated was significantly higher than non-CM-using respondents (30.8% vs. 23.4%, p<0.001, Chi-square test).

### The prevalence and characteristics of CM use

Direct observation showed a prevalence of 37.5% (671/1788) of the concomitant use. Re-calculation using maximum likelihood estimation yielded a similar prevalence of 36.4% (95% CI: 34.2%–38.6%). One hundred and twenty different CM materials used were identified: 92 herbal materials, 12 mineral materials, and 16 animal materials. But 33.7% of CM-using subjects were unable to provide full information about their CM formulae or prescriptions for identifying individual CMs. Only a small portion (6.4%, 43/671) of CM-using patients used single-herbal preparations.

There were 86% of CM-users who used CM therapy initially in order to enhance antipsychotic efficacy, reduce antipsychotic-induced adverse side effects and comorbid psychiatric symptoms (mainly anxiety, depression, cognitive and sleep problems). Sixty-six percent of CM users reported that CM use was recommended by their psychiatrists. Most CMs were prescribed by CM practitioners. Nearly 47% of CM-users were entirely unaware of potential risks of concomitant use of herbal and antipsychotic agents; only 16.4% realized such potential risks. In non-CM-using patients, 35.1% did not know much about CM and 58.1% did not think CM was helpful for their conditions, while only 5.1% were aware of the potential risks of the concomitant treatment.

### Demographic and clinical correlates of CM use

Bivariate analysis displayed significant associations of CM use with gender (p = 0.002), household income (p = 0.001), the illness duration (p<0.001), number of episode (p<0.001), and number of hospitalization (p<0.001) (Table 1). Multivariate analysis further revealed that CM use was significantly associated with male (OR = 1.32, 95% CI 1.09-1.62, p = 0.006), residence in rural areas (OR = 1.48, 95% CI 1.18–1.85, p = 0.001), average (OR = 1.56, 95% CI 1.19–2.06, p = 0.001) and high (OR = 2.36, 95% CI 1.58–3.53, p<0.001) household income, and greater than one year of illness duration (OR = 1.95, 95% CI 1.45–2.63, p<0.001).

**Table 1:** Bivariate and multivariate associations of CM use with demographic and clinical variables in patients with schizophrenia under antipsychotic medication.^a^

Variable	bivariate			Multivariate	
	n	CM use (%)	p	OR (95% CI)	p
Gender			0.002		
Female	879	33.9		1	
Male	909	41.0		1.32 (1.09–1.62)	0.006
Age, yrs			0.108		
<18	92	31.5		1	
18–45	1478	37.1		1.04 (0.58–1.37)	0.899
>45	218	43.1		1.17 (0.71–1.94)	0.540
Marital status			0.109		
Single/divorce/widow	1004	35.9		1	
Married	784	39.7		1.06 (0.86–1.32)	0.589
Education, yrs			0.391		
≤10	1064	36.8		1	
11–13	458	40.2		1.27 (0.99–1.63)	0.062
≥14	266	36.1		1.01 (0.72–1.42)	0.941
Occupation			0.645		
Unemployed	368	38.9		1	
Non-professional	927	37.9		0.92 (0.68–1.24)	0.568
Professional and students	493	35.9		0.81 (0.62–1.08)	0.153
Resident areas			0.135		
Urban	1120	36.2		1	
Rural	668	39.8		1.48 (1.18–1.85)	0.001
Household income b			0.001		
Low	330	30.6		1	
Average	1242	37.9		1.56 (1.19–2.06)	0.001
High	216	45.8		2.36 (1.58–3.53)	<0.001
Diagnostic subtype			0.143		
Paranoid	942	39.2		1	
Non-paranoid c	846	35.7		0.89 (0.73–1.09)	0.245
Duration of the illness, yrs			<0.001		
≤1	396	25.0		1	
>1	1392	41.1		1.95 (1.45–2.63)	<0.001
Number of episodes			<0.001		
≤2	895	32.2		1	
>2	893	42.9		1.14 (0.84–1.53)	0.402
Number of hospitalization			<0.001		
≤2	1142	33.9		1	
>2	646	44.0		1.18 (0.88–1.58)	0.270

a. CM, Chinese medicine. Data analyses were based on CM users (n = 671) with non-CM users (n = 1117) as reference. Chi-square test was used for bivariate analysis and binary logistic regression mode for multivariate analysis.

b. Household income was compared to local average levels.

c. Non-paranoid psychosis includes disorganized, undifferentiated, residual, and tonic types of schizophrenia and schizoaffective disorder.

### CM use correlates of clinical outcomes

While 61.1% (410/671) of CM-using patients and 34.3% (383/1117) of non-CM-using patients displayed improved outcomes, 7.2% (48/671) CM users and 4.4% (49/1117) of non-CM users experienced worse outcomes in the preceding one month (Table 2). Chi-square tests showed that clinical outcomes were significantly associated with whether CM was concomitantly used with antipsychotic drugs (p<0.001). In a binary logistic regression model, CM users had significantly greater odds of improved (OR = 3.44, 95% CI 2.80–4.24, p<0.001) and worse outcomes (OR = 3.15, 95% CI 2.06–4.83, p<0.001) compared to non-CM users.

**Table 2:** Bivariate and multivariate associations between clinical outcomes and CM use in patients with schizophrenia.^a^

Outcomes	Bivariate			Multivariate	
	n	CM users (%)	p	OR (95% CI) b	p
			<0.001		
Unchanged	898	23.7		1	
Improved	793	51.7		3.44 (2.80–4.24)	<0.001
Worse	97	49.5		3.15 (2.06–4.83)	<0.001

a. CM, Chinese medicine. Data analyses were based on CM users (n = 671) with non-CM users (n = 1117) as reference. Chi-square test was used for bivariate analysis and binary logistic regression for multivariate analysis.

b. Binary logistic regression analysis was adjusted for sex, resident areas, household income, duration of illness, number of episode, and number of hospitalization, variables significantly associated with CM use as shown in Table 1.

### Demographic and clinical correlates of clinical outcomes in CM users

Chi-square tests showed that clinical outcomes were significantly associated with resident areas (p<0.001), diagnostic types (p<0.001), and duration of CM use (p = 0.007). Multinomial logistic regression analysis further revealed significant associations of improved outcomes with residence in urban areas (OR = 4.81, 95% CI 3.14–7.36, p<0.001), paranoid schizophrenia (OR = 2.65, 95% CI 1.83–3.84, p<0.001) and more than 3 months of CM use (OR = 1.35, 95% CI 0.91–1.98, p = 0.036). In CM users who experienced worse conditions, similar significant multivariate associations were also observed with residence in urban areas (OR = 6.91, 95% CI 3.09–15.43, p<0.001), paranoid schizophrenia (OR = 1.89, 95% CI 0.96–3.71, p = 0.012), and more than 3 months of CM use (OR = 3.28, 95% CI 1.44–7.46, p = 0.005) (Table 3).

**Table 3:** Bivariate and multivariate associations of clinical outcomes with demographic and clinical variables in schizophrenic patients in Chinese medicine and antipsychotic concomitant treatment.^a^

	Bivariate				Multivariate-IM		Multivariate-WS	
	n	IM (%)	WS (%)	p	OR (95% CI)	p	OR (95% CI)	p
Gender				0.344				
Male	373	62.2	8.0		1		1	
Female	298	59.7	6.0		0.77 (0.53–1.12)	0.173	0.55 (0.28–1.10)	0.091
Age, yrs				0.625				
<18	29	72.4	6.9		1		1	
18–45	548	61.1	7.3		0.76 (0.24–2.43)	0.304	0.73 (0.09–5.81)	0.325
>45	74	57.5	6.4		0.40 (0.14–1.16)	0.231	0.66 (0.10–4.19)	0.438
Marital status				0.349				
Single/divorce/widow	360	58.9	6.9		1		1	
Married	311	63.7	7.4		1.23 (0.83–1.82)	0.816	1.30 (0.63–2.65)	0.478
Education, yrs				0.270				
≤10	391	62.7	8.4		1		1	
11–13	184	58.7	4.9		0.72 (0.46–1.12)	0.183	0.52 (0.21–1.24)	0.299
≥14	96	59.4	6.3		0.92 (0.50–1.69)	0.476	0.90 (0.30–2.73)	0.335
Occupation				0.345				
Unemployed	143	55.2	8.4		1		1	
Non-professional	351	63.3	7.7		1.69 (1.03–2.78)	0.183	1.39 (0.58–3.32)	0.186
Professional/students	177	61.6	5.1		1.17 (0.68–2.02)	0.835	0.70 (0.25–1.93)	0.460
Resident areas				<0.001				
Rural	266	48.9	8.2		1		1	
Urban	405	69.1	5.6		4.81 (3.14–7.36)	<0.001	6.91 (3.09–15.43)	<0.001
Household income b				0.302				
Low	101	56.4	11.9		1		1	
Average	471	62.6	6.4		1.57 (0.74–3.29)	0.213	0.66 (0.18–2.41)	0.191
High	99	58.6	6.1		1.41 (0.82–2.42)	0.136	0.44 (0.18–1.06)	0.291
Diagnostic subtype				<0.001				
Non-paranoid b	302	50.7	7.2		1		1	
Paranoid	369	69.7	7.0		2.65 (1.83–3.84)	<0.001	1.89 (0.96–3.71)	0.012
Duration of illness, yrs				0.991				
≤1	99	60.6	7.1		1		1	
>1	572	61.2	7.2		1.02 (0.56–1.84)	0.958	1.17 (0.40–3.42)	0.769
Number of episodes				0.747				
≤2	288	60.1	8.0		1		1	
>2	383	61.9	6.5		1.13 (0.65–1.97)	0.663	0.75 (0.27–2.13)	0.594
Number of hospitalization				0.780				
≤2	387	61.8	7.5		1		1	
>2	284	60.2	6.7		0.88 (0.51–1.52)	0.655	0.86 (0.31–2.40)	0.775
Duration of CM use, months				0.007				
1–3	233	58.4	3.9		1		1	
>3	438	62.6	8.9		1.35 (0.91–1.98)	0.036	3.28 (1.44–7.46)	0.005

a. CM, Chinese medicine; IM, improved; WS, worse. Data analyses were based on CM users (N = 671) who had clinical outcomes with improved (n = 410), worse (n = 48), and unchanged (n = 213) conditions. The unchanged condition served as reference. Chi-square test was used for bivariate analysis and multinomial logistic regression for multivariate analysis.

b. The definitions of household income and non-paranoid schizophrenia are the same as Table 1.

### Antipsychotic medication correlates of clinical outcomes in CM users

Five different antipsychotic agents and five antipsychotic treatment regimens that were used in at least 5% of CM-using patients with either improved or worse outcomes were identified (Table 4). Multinomial logistical regression analyses, with adjustment for resident areas, diagnostic types, and duration of CM use, variables that were significantly associated with clinical outcomes, revealed no significant associations with any antipsychotic treatment regimens favoring improve outcomes, but significantly lower odds of improved outcomes were observed in patients whose antipsychotic regimens included olanzapine (OR = 0.48, 95% CI 0.27–0.85, p = 0.035). There were significantly higher odds of worse outcomes in subjects whose antipsychotic regimens included quetiapine (OR = 1.90, 95% CI 0.88–4.08, p = 0.013), quetiapine alone (OR = 2.16, 95% CI 0.95–4.95, p = 0.031) or clozapine alone (OR = 3.02, 95% CI 0.94–9.70, p = 0.025) (Table 4).

**Table 4:** Multivariate associations between clinical outcomes and antipsychotic medication modes in patients with schizophrenia who were treated concomitantly with CM.^a^

	Improved (n = 410)			Worse (n = 48)		
	%	OR (95% CI)	p	%	OR (95% CI)	p
The most frequently usedantipsychotics						
Risperidone	56.1	1.22 (0.86–1.75)	0.900	41.7	0.81 (0.42–1.56)	0.696
Quetiapine	19.5	0.75 (0.49–1.16)	0.463	27.1 b	1.90 (0.88–4.08)	0.013
Clozapine	20.7	1.40 (0.90–2.19)	0.542	22.9	1.29 (0.63–2.66)	0.068
Olanzapine	7.1	0.48 (0.27–0.85)	0.035	12.5	0.97 (0.37–2.54)	0.463
Phenothiazines	6.5	0.92 (0.47–1.81)	0.819	2.1	0.25 (0.03–1.95)	0.837
The most frequently usedantipsychotic regimens						
Risperidone alone	33.9	1.34 (0.90–1.99)	0.276	33.3	1.14 (0.56–2.31)	0.424
Quetiapine alone b	11.7	0.69 (0.41–1.17)	0.063	27.1 b	2.16 (0.95–4.95)	0.031
Clozapine + risperidone	6.3	1.43 (0.65–3.17)	0.527	8.3	2.63 (0.74–9.38)	0.064
Clozapine alone	4.6	1.37 (0.60–3.12)	0.438	12.5	3.02 (0.94–9.70)	0.025
Olanzapine alone	4.1	0.57 (0.27–1.22)	0.213	12.5	1.88 (0.66–5.34)	0.399

a. CM, Chinese medicine. Unchanged outcomes (n = 213) served as reference. Multinomial logistical regression analysis was adjusted for resident areas, diagnostic subtype, and duration of CM use, variables significantly associated with clinical outcomes as shown in Table 3.

b. It is noted that all quetiapine-including treatment regimens are quetiapine monotherapy.

### Individual CM correlates of clinical outcomes

Of 120 different CM materials identified, 21 (20 herbal materials and one animal material) were used in at least 5% of CM-using subjects with either improved or worse outcomes (Table 5). Multinomial logistic regression model analysis showed no significant associations of improved outcomes with any individual CMs identified, but the inclusion of Acorus gramineus in CM treatment regimens significantly reduced odds of worse outcomes compared to improved outcomes (OR = 0.11, 95% CI 0.01–0.88, p = 0.037). Significantly higher odds of worse outcomes were observed in subjects whose CM treatment regimens included Radix Bupleuri (OR = 2.49, 95% CI 1.10–5.62, p = 0.028), Fructus Gardenia (OR = 9.16, 95% CI 4.19–20.02, p<0.001), Fructus Schisandrae (OR = 3.90, 95% CI 1.42–10.73, P = 0.008), Radix Rehmanniae (OR = 3.48, 95% CI 1.03–11.70, p = 0.044), Akebia Caulis (OR = 14.6, 95% CI 2.50–31.87, p<0.001), and Semen Plantaginis (OR = 21.10, 95% CI 4.32–103.05, p<0.001). Frequency distributions revealed that concomitant treatment regimens containing these six herbal materials and worse outcome-associated antipsychotics accounted for 59.8% (49/82) of total identified concomitant treatment regimens in patients with worse outcomes (Table 6).

**Table 5:** Multivariate associations of clinical outcomes with individual CMs in concomitant use with antipsychotics in schizophrenic patients.^a^

Individual Chinese medicine	Improved (n = 410)			Worse (n = 48)		
	%	OR (95% CI)	p	%	OR (95% CI)	p
Radix Glycyrrhizae (Gan-Cao)	26.8	1.59 (1.06–2.38)	0.232	12.5	0.53 (0.21–1.35)	0.181
Acorus gramineus (Shi-Chang-Pu)	17.3	1.11 (0.66–1.87)	0.699	2.1	0.11 (0.01–0.88)	0.037
Ziziphus jujuba (Suan-Zao-Ren)	14.1	0.63 (0.39–1.01)	0.056	18.8	0.83 (0.36–1.90)	0.660
Curcuma root (Yu-Jin)	13.9	1.76 (0.98–3.17)	0.058	14.6	1.84 (0.70–4.79)	0.214
Radix Angelica Sinensis (Dang-Gui)	13.9	1.03 (0.63–1.70)	0.901	8.3	0.63 (0.21–1.92)	0.422
Poria cocos (Fu-Ling)	13.2	1.30 (0.76–2.24)	0.335	12.5	1.28 (0.48–3.40)	0.620
Radix Bupleuri (Chai-Hu)	12.2	1.14 (0.68–1.93)	0.616	22.9	2.49 (1.10–5.62)	0.028
Radix Polygalae (Yuan-Zhi)	10.0	0.86 (0.50–1.49)	0.599	20.8	1.82 (0.77–4.31)	0.173
Salvia Miltrorrhiza (Dan-Shen)	9.8	0.87 (0.49–1.53)	0.625	10.4	0.92 (0.33–2.61)	0.881
Lumbricus (Di-Long)	9.8	0.95 (0.54–1.68)	0.863	4.2	0.17 (0.02–1.30)	0.088
Rhizoma Atractylodes (Bai-Zhu)	9.8	1.31 (0.69–2.47)	0.408	4.2	0.51 (0.11–2.34)	0.388
Dried tangerine peel (Chen-Pi)	9.5	1.16 (0.63–2.12)	0.635	8.3	0.94 (0.30–2.97)	0.921
Fructus Gardenia (Zhi-Zi)	8.3	1.02 (0.55–1.90)	0.947	45.8	9.16 (4.19–20.02)	<0.001
Flos Carthami (Hong-Hua)	8.3	0.99 (0.57–1.75)	0.984	4.2	0.47 (0.11–2.08)	0.318
Rhizoma Chuan Xiong (Chuan-Xiong)	7.6	0.73 (0.40–1.33)	0.306	4.2	0.38 (0.09–1.69)	0.204
Semen Persicae (Tao-Ren)	7.3	0.92 (0.49–1.75)	0.808	4.2	0.53 (0.12–2.41)	0.413
Magnolia officinalis (Hou-Po)	4.3	0.59 (0.26–1.30)	0.190	8.3	1.05 (0.31–3.52)	0.938
Fructus Schisandrae (Wu-Wei-Zi)	4.1	0.89 (0.38–2.07)	0.782	22.9	3.90 (1.42–10.73)	0.008
Radix Rehmanniae (Di-Huang)	3.4	1.22 (0.49–3.06)	0.669	12.5	3.48 (1.03–11.70)	0.044
Akebia Caulis (Mu-Tong) b	1.0	0.49 (0.12–2.04)	0.326	25.0	8.92 (2.50–31.87)	<0.001
Semen Plantaginis (Che-Qian-Zi)	0.7	1.64 (0.17–16.20)	0.671	33.3	21.10 (4.32–103.05)	<0.001

a. CM, Chinese medicine. The unchanged outcomes (n = 213) served as reference. Models were adjusted for resident areas, diagnostic subtype, and duration of CM use, variables significantly associated with clinical outcomes as shown in Table 3.

b. Akebia Caulis includes the two species: Akebia quinata (Thunb.) Decne. and Akebia trifoliate (Thunb.) Koidz.

**Table 6:** Frequency distribution of herbal and antipsychotic concomitant treatment regimens associated with adverse outcome in schizophrenic patients.

	Risperidone alone	Quetiapine alone a,b	Clozapine alone a	Olanzepine-including a	Risperidone +clozapine	Others
Radix Bupleuri	4	1	3	2	1	0
Fructus Gardenia	7	7	2	3	3	0
Fructus Schisandrae	4	4	2	1	0	0
Radix Rehmanniae	2	2	2	0	0	0
Akebia Caulis	4	5	1	1	1	0
Semen Plantaginis	3	7	3	3	0	0
Others c	2	0	0	1 c	0	1
**Subtotal**	**26**	**26**	**13**	**11**	**5**	**1**
		**49**				
**Total**	**82**					

a. These antipsychotic regimens are significantly associated with adverse outcome as shown in Table 4.

b. It is noted that all quetiapine-including treatment regimens are quetiapine monotherapy.

c. Other herbal material-including regimens were not counted in subtotal regimens associated with adverse outcomes.

## Discussion

In our survey of a representative sample of patients with schizophrenia, nearly 36% of them had concomitant CM and antipsychotic treatment. This prevalence rate of CM use is somewhat lower than that observed in other commonly occurring chronic conditions in Chinese communities [17]–[19]. In non-CM-using patients, nearly 58% did not believe CM could help their condition, suggesting that the lower prevalence of CM use in schizophrenic population is mainly related to their negative attitude towards this traditional remedy. Unlike patients in Western society where a minority of them informed their doctors of their use of alternative medicine [20], [21], CM use in most patients in this study was recommended by their psychiatrists. However, only about one-third of patients were aware of the potential risks of concomitant treatment with CM. These findings may reflect an underestimation of both potential benefits and risks of CM use among patients and their psychiatrists.

We found that concomitant CM use was significantly associated with male, residence in rural areas, relatively higher household income, longer duration of illness, and more episodes and hospitalizations. These associations suggest that the men living in rural areas may have greater positive beliefs about CM for their illnesses. Previous studies also showed that people who had persistent and recurrent mental-emotional problems more often sought alternative therapies than populations with other chronic diseases [21], [22].

While it was similar in most antipsychotic treatment regimens in CM- and non-CM-using patients, there exist significant bivariate and multivariate associations between CM use and clinical outcomes. Patients in CM concomitant treatment had a significantly greater chance of improved clinical outcomes compared to non-CM use (61.1% vs. 34.3%, OR = 3.44, 95% CI 2.80–4.24). However, a small but significant number of patients treated concomitantly with CM had a greater risk of developing worse outcomes (7.2% vs. 4.4%, OR = 2.06, 95% CI 2.06–4.83). These data clearly indicate that CM concomitant treatment could produce either beneficial or adverse effects on clinical outcome, probably depending on different combinations of CM and antipsychotics.

Furthermore, bivariate and multivariate analyses of CM-using subgroup revealed that both improved and worse outcomes were significantly associated with residence in urban areas, suggesting that patients living in urban areas may have greater impacts with CM therapy than those in rural areas. This is likely due in part to the fact that urban patients generally have more unconventional and conventional treatment options compared to rural patients [16]. This perhaps results in an increase in unpredictable positive and negative clinical effects, as the therapeutic properties of most CM preparations are not yet well identified. Meanwhile, the addition of CM significantly increased the chances of improved outcomes in paranoid patients compared to non-paranoid subtype. Several studies have demonstrated differences in neuropsychological character and clinical response to antipsychotic treatment between paranoid and non-paranoid subtypes [23], [24] as well as subtype specificity of genetic profile [25], [26]. Thus, the greater chance of alteration in treatment outcomes observed in paranoid patients may reflect a similar subtype difference in clinical effects of CM treatment.

Our results demonstrated that exceeding 3 months of CM use is a significant predictor for both improved and worse clinical outcomes. This finding confirms empirical evidence, suggesting that CM therapy of most chronic conditions requires a considerable duration in order to achieve observable improvement [14]. However, the finding is also consistent with those of case studies, revealing that most nephrotoxic and hepatotoxic effects associated with herbal medicine use are observed after 1–5 months of intake [4], [5], [27]. Considering difficulties in monitoring herbal toxicity and potential herb-drug interactions due to complex mixtures of unknown and unidentified ingredients in CM, the determination of an optimal length of the treatment might be a feasible strategy in minimizing adverse and toxic effects while maximizing beneficial effects. For this purpose, correlations between different length of CM use and changes in pharmacokinetic profile of conventional drugs may deserve to be further determined.

We found that CM-using patients whose antipsychotic regimens included olanzapine had significantly lower chance of improved outcomes. Meanwhile, quetiapine and clozapine monotherapy significantly heightened the risk of developing worse outcomes, suggesting associations of these three atypical antipsychotics with adverse clinical outcomes when used concomitantly with CM. On the other hand, among seven individual CM materials identified to be significantly associated with clinical outcomes, six were found to be significantly associated with adverse outcomes. They were Radix Bupleuri, Fructus Gardenia, Fructus Schisandrae, Radix Rehmanniae, Akebia Caulis, and Semen Plantaginis. Moreover, the concomitant treatment regimens including these herbal materials and antipsychotics associated with adverse outcomes accounted for nearly 60% of total identified treatment regimens in patients with worse outcomes. These data suggest that the heightened risk of adverse outcomes observed is closely associated with these herbal agents in combination with antipsychotic regimens.

As the identified herbal medicines have been well demonstrated to have high safety profiles [28], the adverse outcomes observed seem to be attributable to herb-drug pharmacokinetic interactions in which the pattern of drug metabolism is altered. Despite lack of information about the interactions between herbal and antipsychotic agents, early case studies reported that ginseng combined with phenelzine and betel nut with fluphenazine caused broad adverse effects in schizophrenic patients [29]–[31]. Our recent study of bipolar patients also found that combination treatment with the mood stabilizer carbamazepine and an herbal preparation for 26 weeks resulted in a significantly lower level of serum carbamazepine compared to carbamazepine alone, suggesting that the addition of herbal medicine accelerates carbamazepine metabolism and lowers its blood concentration [32]. Like carbamazepine, most antipsychotic drugs, including clozapine, olanzapine, and quetiapine, are metabolized as substrates for cytochrome P450s (CYPs) [33], [34]. Therefore, pharmacokinetic interactions may play an important role in influencing clinical outcomes in patients with schizophrenia under concomitant treatment of herbal and antipsychotic agents observed in the present study.

There are several limitations in the present study. First, as patients who sought CM treatment may have distinctive perceptions about herbal medicine, psychological or “placebo” factors could not be excluded. The use of structured assessment instruments, including symptom scales and laboratory tests, should be helpful in further clarifying treatment effects of CM therapy. Second, since a considerable portion of CM-users were unable to provide full information about their CM formulae, it may lead to an underestimation of individual CMs associated with clinical outcomes. On the other hand, due to difficulties in collecting information about dosages of herbal and antipsychotic agents as well as the quality of herbal preparations, these factors were not considered in the present study. However, it should be noted that there have been extensive reports about severe adverse events caused by overdosing, heavy metal contaminations, and adulterants with conventional drugs of herbal supplements [5], [35], [36], all of which might account for the presumed adverse effects of herbal and antipsychotic combinations. Fourth, as the majority of CM treatments were recommended by psychiatrists to their patients, obtaining information about psychiatrists' attitudes and knowledge of CM would be helpful in devising safe and effective strategies of concomitant CM and antipsychotics in the treatment of schizophrenia. Finally, although there are many statistically significant results found in the study, “chance significances” may not be excluded. Additional cautions should be paid when the results are considered with reference in future studies.

In conclusion, a relatively small proportion of patients with schizophrenia have concomitant CM and antipsychotic treatment. Such concomitant treatment may heighten the risk of developing worse clinical outcomes in a small number, but increase the chance of improving treatment outcomes in a much greater number of patients. Better identification of the concomitant herbal and antipsychotic treatment regimens that are associated with clinical outcomes provides useful hints for further clarifying herb-drug pharmacokinetic interactions.
